# The Natural Janus Kinase Inhibitor Agerarin Downregulates Interleukin-4-Induced *PER2* Expression in HaCaT Keratinocytes

**DOI:** 10.3390/molecules27134205

**Published:** 2022-06-30

**Authors:** Jeong Yeon Lee, Euitaek Jung, Hyunjin Yeo, Sung Shin Ahn, Yoongho Lim, Young Han Lee

**Affiliations:** 1 Department of Biological Sciences, Sanghuh College of Lifesciences, Konkuk University, Seoul 05029, Korea; wjddusdl3010@naver.com (J.Y.L.); mylife4sci@konkuk.ac.kr (E.J.); jini1606@konkuk.ac.kr (H.Y.); wendy713@konkuk.ac.kr (S.S.A.); 2 Division of Bioscience and Biotechnology, BMIC, Konkuk University, Seoul 05029, Korea; yoongho@konkuk.ac.kr; 3 Cancer and Metabolism Institute, Konkuk University, Seoul 05029, Korea

**Keywords:** agerarin, circadian rhythm, interleukin-4, Janus kinase, PER2, STAT3

## Abstract

The circadian clock system is closely associated with inflammatory responses. Dysregulation of the circadian clock genes in the skin impairs the skin barrier function and affects the pathophysiology of atopic dermatitis. Interleukin 4 (IL-4) is a proinflammatory cytokine derived from T-helper type 2 cells; it plays a critical role in the pathogenesis of atopic dermatitis. Agerarin (6,7-dimethoxy-2,2-dimethyl-2*H*-chromene) is a natural JAK1/2/3 inhibitor isolated from *Ageratum houstonianum* that has a protective effect on the epidermal skin barrier. However, it remains unclear whether agerarin affects the circadian clock system. The aim of this study is to investigate the effect of agerarin on IL-4-induced *PER2* gene expression in human keratinocytes through reverse transcription (RT)-PCR, quantitative real-time PCR (qPCR), immunoblotting, immunofluorescence microscopic analysis, and real-time bioluminescence analysis. We found that agerarin reduced IL-4-induced *PER2* mRNA expression by suppressing the JAK-STAT3 pathway. In addition, real-time bioluminescence analysis in PER2:luc2p promoter-reporter cells revealed that agerarin restored the oscillatory rhythmicity of *PER2* promoter activity altered by IL-4. These findings suggest that agerarin may be useful as a cosmeceutical agent against inflammatory skin conditions associated with disrupted circadian rhythms, such as atopic dermatitis.

## 1. Introduction

The circadian rhythm is an endogenous oscillating system that controls various biological and physiological processes with an approximately 24 h cycle [1]. The central clock in mammals is located in the suprachiasmatic nucleus (SCN) of the hypothalamus and acts as an essential circadian pacemaker. Most peripheral tissues are synchronized by the central clock, transmitting periodic signals, such as hormones, metabolites, and neuronal signals. The circadian clock is regulated by core clock genes, including brain and muscle aryl hydrocarbon receptor nuclear translocator-like 1 (*BMAL1*), circadian locomotor output cycles kaput (*CLOCK*), period circadian regulator (*PER*)1/2, cryptochromes (*CRY*), retinoic acid-related orphan receptor alpha, and nuclear receptor subfamily 1 group D member 1 (*NR1D1*, also known as REV-ERBα/β) [2]. At the molecular level, circadian rhythms are generated by oscillating clock-related genes that organize a transcription–translation feedback loop [3]. The core circadian loop includes transcriptional activators, such as BMAL1 and CLOCK complexes, which drive the expression of PER1/2 and CRY by binding to E/E’-box elements present in their promoter region. PER1/2 and CRY complexes, in turn, inhibit BMAL1 and CLOCK complex-mediated transcription, thereby negatively regulating their own transcription [4].

Approximately 10% of the total genome can be regulated by the core circadian clock genes in every organ, including the skin [4,5,6,7]. The skin is the organ most susceptible to external environments, such as ultraviolet (UV) radiation, humidity, pathogens, and temperature. Various functions of the human skin, including transepidermal water loss, sebum production, cell proliferation, pH and temperature regulation, and itch, undergo circadian rhythm variations [4,8]. Human skin cells, including keratinocytes, melanocytes, and fibroblasts, are known to express various functional circadian clock genes [9,10,11]. Dysregulation of the circadian clock genes in the skin impairs its barrier function and affects the pathophysiology of inflammatory diseases, such as atopic dermatitis (AD) and psoriasis [12,13,14].

The circadian clock controls immune functions, and inflammatory cytokines substantially affect circadian rhythms at the molecular, cellular, and behavioral levels [15,16,17,18,19,20]. Indeed, depletion of *Bmal1* facilitates lipopolysaccharide (LPS)-induced interleukin (IL)-1β production [21], and deletion of *Cry1* increases LPS-induced IL-6 and tumor necrosis factor alpha (TNFα) expression [22]. Based on these studies, it is suggested that abnormal expression of core clock genes may contribute to the pathophysiology of skin inflammation. Conversely, inflammatory cytokines modulate the expression of circadian clock genes [23,24]. IL-4 is a cytokine secreted by T-helper type 2 cells (Th2), which are overexpressed in AD skin lesions and play a critical role in the pathogenesis of AD [25]. PER2 is necessary for circadian clock rhythm resetting [26] and regulation of rhythmic period length [27]. However, it is unclear whether IL-4 regulates PER2 expression and alters the circadian clock system in keratinocytes.

Agerarin (6,7-dimethoxy-2,2-dimethyl-2*H*-chromene) is a bioactive compound identified in the ethanolic extract of *Ageratum houstonianum* (AHE) that functions as a Janus kinase (JAK)-1, -2, and -3 inhibitor [28] and induces CLOCK expression to upregulate aquaporin-3 (AQP3), a membrane water transporter protein, in human keratinocytes [29]. However, it remains unclear whether agerarin affects IL-4-induced PER2 expression.

This study aimed to investigate whether agerarin affects *PER2* gene expression in human keratinocytes. In this study, we used a transformed epidermal keratinocyte cell line, HaCaT, which is derived from normal adult skin. This cell line closely reflects keratinocyte cell behavior and possesses functional epidermal physiology controlled by the circadian clock, similar to neonatal foreskin keratinocytes [30]. Here, we show that IL-4 upregulates *PER2* expression at the mRNA level and that agerarin reduces IL-4-induced *PER2* expression by inhibiting the JAK/signal transducer and activator of the transcription 3 (STAT3) signaling pathway. Furthermore, we observed that agerarin restores the IL-4-induced amplitude of the circadian rhythmicity of *PER2* promoter activity, as revealed by live-cell bioluminescence.

## 2. Results

### 2.1. IL-4 Upregulates PER2 Expression in HaCaT Keratinocytes

**Figure 1:**
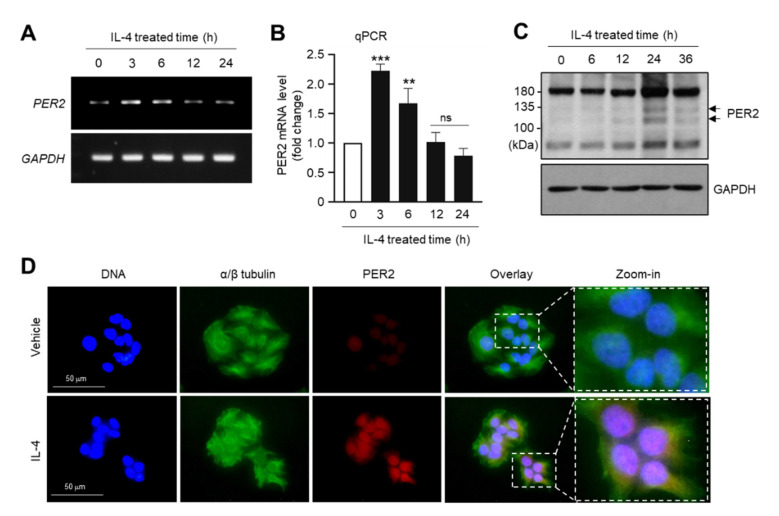
Effect of IL-4 on the expression of PER2. HaCaT cells were treated with vehicle (PBS) or 20 ng/mL IL-4 for various periods (0–36 h). (**A**,**B**) Total RNA was isolated, and the levels of *PER2* mRNA were measured by RT-PCR (**A**) and qPCR (**B**). The mRNA level of *GAPDH* was used as an internal control. ns, not significant; ** *p* < 0.01; *** *p* < 0.001 compared to vehicle (PBS)-treated control group (*n* = 3) by Sidak’s multiple comparisons test. (**C**) Whole-cell lysates were prepared and immunoblotted using anti-IL31 antibodies. GAPDH was used as the loading control. (**D**) HaCaT cells cultured on coverslips were treated with vehicle (PBS) or 20 ng/mL IL-4 for 24 h, followed by fixing and permeabilization. Immunofluorescence staining was performed using anti-PER2 and Alexa Fluor 555-conjugated secondary antibodies (red staining). α/β-tubulin was counterstained with anti-α/β tubulin and Alexa Fluor 488-conjugated secondary antibodies (green staining). Nuclear DNA was stained with 1 μg/mL Hoechst 33258 (blue staining). Fluorescent cells were captured with an EVOSf1 fluorescence microscope. The last panel on the right is a zoomed-in view of the dotted box. Scale bars, 50 μm. IL-4, interleukin 4; GAPDH, glyceraldehyde 3-phosphate dehydrogenase; PBS, phosphate-buffered saline; PER2, Period2; RT-PCR, reverse transcription polymerase chain reaction; qPCR, quantitative real-time PCR.

We examined the effect of IL-4 on PER2 expression in HaCaT keratinocytes. The cells were treated with 20 ng/mL IL-4, and *PER2* mRNA expression was measured. Reverse transcription (RT)-PCR analysis revealed that *PER2* mRNA expression peaked 3 h after IL-4 stimulation in HaCaT cells (Figure 1A). Quantitative real-time PCR (qPCR) demonstrated an approximately 2.1-fold increase in *PER2* mRNA levels relative to control levels after 3 h of treatment (Figure 1B). Immunoblot analysis demonstrated an increase in the level of PER2 protein after 24 h of IL-4 stimulation (Figure 1C). Immunofluorescence staining showed that IL-4-induced PER2 proteins were localized in the nucleus of the cells (Figure 1D). These data suggest that IL-4 upregulates *PER2* expression at the mRNA level in HaCaT keratinocytes.

### 2.2. The JAK/STAT3 Pathway Is Involved in IL-4-Induced PER2 Expression

**Figure 2:**
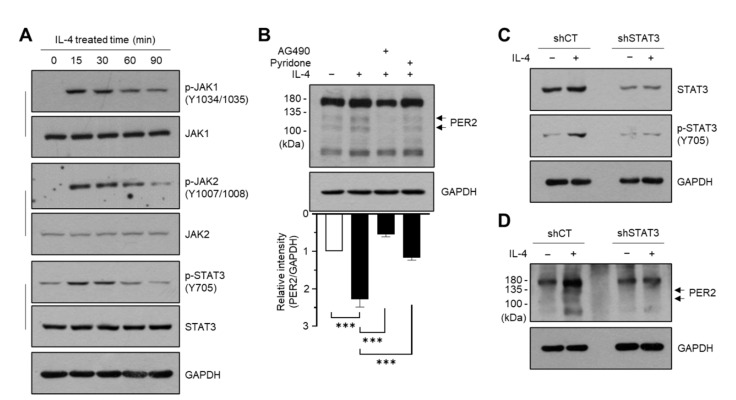
Role of the JAK/STAT3 pathway in IL-4-induced PER2 expression. (**A**) HaCaT cells were treated with 20 ng/mL IL-4 for different periods (0–60 min). Whole-cell lysates were prepared, and immunoblotting was performed using phosphorylation-specific or total protein antibodies. GAPDH was used as an internal control. Uncropped blots are shown in Figure S2. (**B**) HaCaT cells were treated with 20 ng/mL IL-4 for 24 h in the absence or presence of 40 μM AG490 and 6.2 μM pyridone 6. GAPDH was used as an internal control. Relative band intensities were measured using ImageJ. *** *p* < 0.001 compared to IL-4 alone-treated group (*n* = 3) by Sidak’s multiple comparisons test. (**C**,**D**) HaCaT cells expressing scrambled (shCT) or STAT3 shRNA (shSTAT3) were treated with (+) or without (−) 20 ng/mL IL-4 for 15 min (**C**) or 24 h (**D**). The protein levels of p-STAT3 (Tyr705) and total STAT3 (**C**) and PER2 (**D**) were measured by immunoblotting. GAPDH was used as an internal control. JAK, Janus kinase; STAT3, signal transducer and activator of transcription 3.

Upon IL-4 stimulation, activated JAK leads to phosphorylation of STAT, which then translocates to the cell nucleus to regulate target genes [31,32]. Consistent with previous studies, we observed that the phosphorylation of JAK1 at Tyr1034/1035, JAK2 at Tyr1007/1008, and STAT3 at Tyr705 increased within 15 min in response to IL-4 stimulation (Figure 2A).

To investigate whether the JAK/STAT3 pathway is associated with PER2 expression by IL-4 stimulation, we examined the effect of pharmacological JAK inhibitors on IL-4-induced PER2 expression. Pretreatment with 40 μM AG490 (JAK2-specific inhibitor) or 6.2 μM pyridone 6 (pan-JAK inhibitor) significantly inhibited the IL-4-induced accumulation of PER2 (*p* < 0.001; Figure 2B). To further determine the role of the JAK/STAT3 pathway in IL-4-induced PER2 expression, we used stable transfectants of HaCaT cells expressing scrambled shRNA (shCT) or STAT3 shRNA (shSTAT3). Stable knockdown of *STAT3* and suppressed phosphorylation of STAT3 following IL-4 treatment were confirmed by immunoblotting (Figure 2C). Under these experimental conditions, IL-4-induced accumulation of PER2 was considerably reduced in shSTAT3 cells compared to shCT cells (Figure 2D). These data suggest that IL-4 regulates *PER2* expression through the JAK/STAT3 signaling pathway.

### 2.3. Agerarin Inhibits IL-4-Induced JAK/STAT3 Signaling Pathway

**Figure 3:**
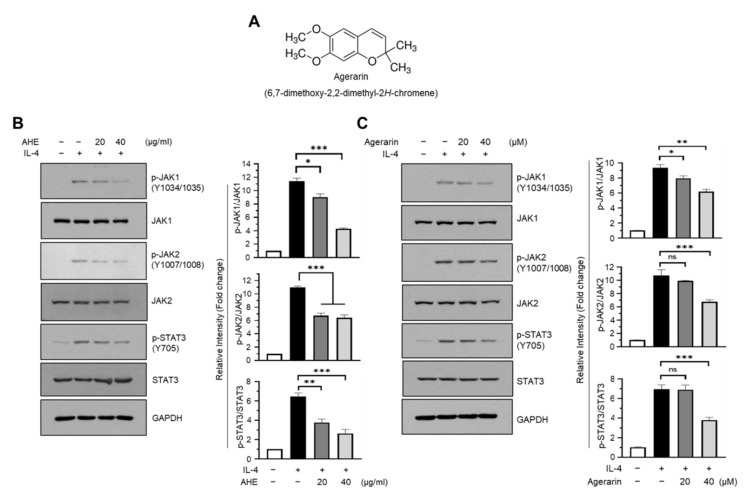
Effect of AHE and agerarin on inhibition of the JAK/STAT3 signaling pathway stimulated by IL-4. (**A**) Chemical structure of agerarin. (**B**,**C**) HaCaT cells were pretreated with 20 and 40 μg/mL AHE (**B**) or 20 and 40 μM agerarin (**C**) for 30 min before stimulation with (+) or without (−) 20 ng/mL IL-4. After 15 min, cells were harvested, and the phosphorylation of JAK1 at Y1034/1035, JAK2 at Y1007/1008, and STAT3 at Y705 were measured by immunoblotting. Band intensities of phosphorylated proteins were measured using ImageJ and normalized to the corresponding total proteins. GAPDH was used as an internal control. ns, not significant; * *p* < 0.05, ** *p* < 0.01, *** *p* < 0.001 compared to control (*n* = 3) by an unpaired two-tailed *t*-test. AHE, ethanolic extract of *Ageratum houstonianum*.

Agerarin (Figure 3A) is an active component of AHE that inhibits JAK1 and JAK2 kinase activities [28]. Therefore, we considered the possibility that agerarin may affect IL-4-induced *PER2* expression through inhibition of JAK1/2 kinase activity. To test this possibility, we first confirmed the effect of AHE and agerarin on the inhibition of the JAK/STAT3 signaling pathway in HaCaT keratinocytes. IL-4-induced phosphorylation of JAK1 at Y1034/1035, JAK2 at Y1007/1008, and STAT3 at Tyr705 were inhibited by AHE (Figure 3B) and agerarin (Figure 3C) in a dose-dependent manner. These data demonstrate that agerarin inhibits IL-4-induced activation of the JAK/STAT3 signaling pathway.

### 2.4. Agerarin Abrogates IL-4-Induced PER2 Expression in HaCaT Cells

**Figure 4:**
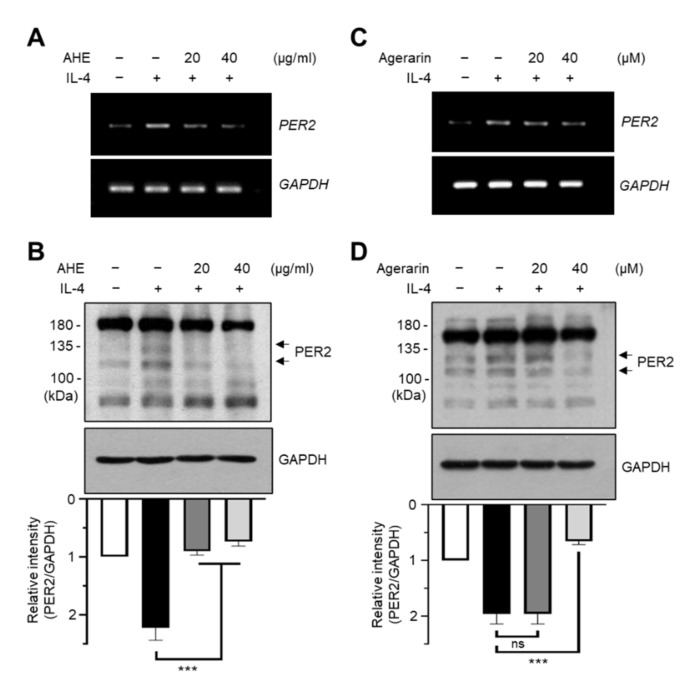
Effect of agerarin and AHE on the suppression of IL-4-induced *PER2* expression. (**A**,**C**) HaCaT cells were pretreated with 20 or 40 μg/mL AHE (**A**) and 20 or 40 μM agerarin (**C**) for 30 min before stimulation in the presence (+) or absence (−) of 20 ng/mL IL-4. After 3 h, total RNA was isolated, and the levels of *PER2* mRNA were measured by RT-PCR. The mRNA level of *GAPDH* was used as an internal control. (**B**,**D**) HaCaT cells were pretreated with AHE (**B**) and agerarin (**D**), as described above. After 24 h, whole-cell lysates were prepared and immunoblotted using anti-PER2 antibodies. GAPDH was used as the loading control. Band intensities of PER2 protein were measured using ImageJ and normalized to GAPDH. ns, not significant; *** *p* < 0.001 compared to IL-4-treated group (*n* = 3) by Sidak’s multiple comparisons test.

**Figure 5:**
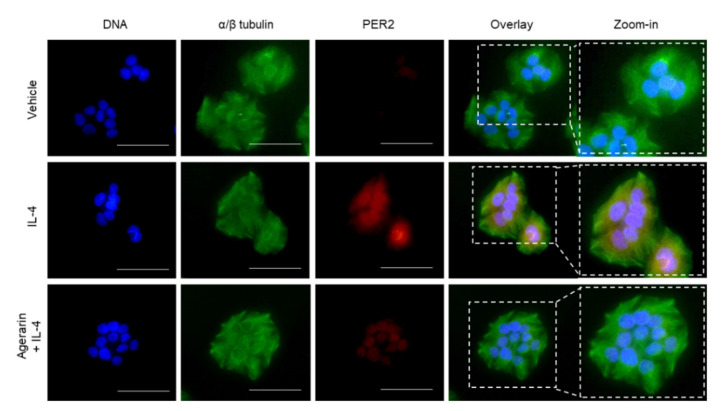
Effect of agerarin on IL-4-induced localization of PER2. HaCaT cells cultured on coverslips were treated with vehicle (PBS), 20 ng/mL IL-4, or 20 ng/mL IL-4 plus 40 μM agerarin for 24 h, followed by fixing and permeabilization. Immunofluorescence staining was performed using anti-PER2 and Alexa Fluor 555-conjugated secondary antibodies (red staining). The α/β-tubulin was counterstained with anti-α/β-tubulin and Alexa Fluor 488-conjugated secondary antibodies (green staining). Nuclear DNA was stained with 1 μg/mL Hoechst 33258 (blue staining). Fluorescent cells were captured with an EVOSf1 fluorescence microscope. The last panel on the right is a zoomed-in view of the dotted box. Scale bars, 50 μm.

To investigate whether JAK1/2 inhibitory agerarin affects IL-4-induced PER2 expression, we pretreated HaCaT cells with AHE and agerarin prior to IL-4 addition. RT-PCR analysis showed that both AHE (Figure 4A) and agerarin (Figure 4B) reduced IL-4-induced *PER2* mRNA levels. In addition, the IL-4-induced increase in PER2 protein levels was also significantly (*p <* 0.001) reduced by pretreatment with AHE (Figure 4C) and agerarin (Figure 4D). Furthermore, immunofluorescence staining showed that the IL-4-induced abundance of nuclear PER2 was reduced by agerarin pretreatment in HaCaT cells (Figure 5). These results demonstrate that agerarin isolated from AHE inhibits IL-4-induced PER2 expression at the mRNA and protein level.

### 2.5. Agerarin Inhibits IL-4-Induced PER2 Expression in HaCaT Cells Synchronized with Dexamethasone

**Figure 6:**
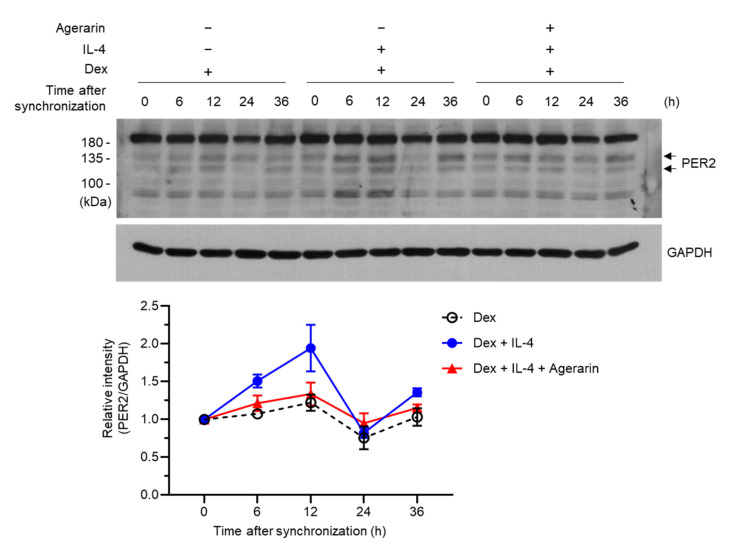
Effect of agerarin on the suppression of IL-4-induced PER2 expression in HaCaT cells synchronized with Dex. HaCaT cells were synchronized with 100 nM Dex for 2 h, followed by the addition of in the presence (+) or absence (−) of 20 ng/mL IL-4 or 20 ng/mL IL-4 plus 40 μM agerarin. After 24 h, whole-cell lysates were prepared and immunoblotted using anti-PER2 antibodies. GAPDH was used as the loading control. Band intensities of PER2 protein were measured using ImageJ and normalized to GAPDH. Dex, dexamethasone.

Treatment with dexamethasone is known to exert a synchronizing effect on clocks in cultured cells through rhythmic expression of several clock genes, such as *PER2* in HaCaT keratinocytes [30]. To further characterize the functional effect of agerarin, we synchronized HaCaT cells with dexamethasone, as reported previously [33], and examined the effect of agerarin on IL-4-induced PER2 expression. As observed in a previous study [30], PER2 expression peaked at 12 h and returned to baseline levels 24 h after synchronization (Figure 6). As predicted, IL-4 stimulation substantially increased the peak, which was reduced by agerarin treatment. Notably, PER2 expression increased again at 36 h, suggesting that it oscillates after synchronization with dexamethasone.

### 2.6. Construction of Live-Cell Bioluminescence Reporter for Oscillation of PER2 Promoter Activity

To further evaluate whether agerarin affects the oscillation of *PER2* expression, we isolated the 5′-regulatory region of the human *PER2* gene, spanning nucleotides −500 to +50, which contains multiple *cis*-acting regulatory elements, including E-box, D-box, GC box elements, circadian transcription enhancing site, transcription start site, and STAT3-binding site (Supplementary Figure S1A). A bioluminescence reporter plasmid, PER2:luc2P encoding the firefly luciferase 2P gene (*luc2P*) under the control of the human *PER2* promoter (−500/+50), was constructed and transfected into HaCaT cells to establish a stable bioluminescent reporter cell line (HaCaT/PER2:luc2P). After synchronizing with 100 nM dexamethasone for 2 h, as reported previously [33], the bioluminescence of PER2:luc2P maintained a circadian oscillation for up to 90 h (Supplementary Figure S1B), suggesting that *PER2* expression displays a circadian rhythmic pattern in synchronized HaCaT cells.

**Figure 7:**
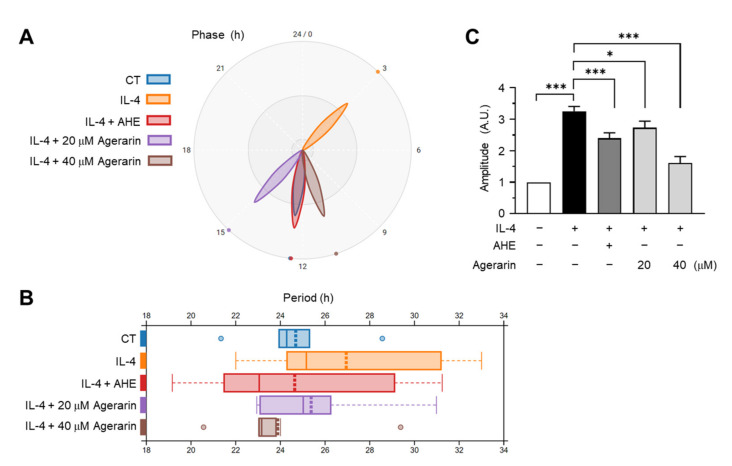
Real-time bioluminescence of the PER2:luc2P reporter. HaCaT/PER2:luc2P cells seeded in 96-well plates were synchronized with 100 µM Dex for 2 h, after which the medium was replaced with the recording phenol-free medium containing 1% fetal bovine serum and 1 mM luciferin. Real-time bioluminescence of the PER2:luc2P reporter was measured and recorded every 20 min for 90 h using Spark10M. (**B**–**D**) After synchronization with 100 nM Dex, HaCaT/PER2:luc2P reporter cells cultured on 96-well plates were treated with 20 ng/mL IL-4 in the presence (+) or absence (−) of 20 μg/mL AHE or agerarin (20 and 40 μM). Real-time bioluminescence of the PER2:luc2P reporter was measured using Spark10M. The phase (**A**), period (**B**), and amplitude (**C**) of the bioluminescence of the PER2:luc2P reporter were analyzed using Biodare2. * *p* < 0.05; *** *p* < 0.001 (*n* = 12) by Sidak’s multiple comparisons test. CT, synchronization only control; A.U., arbitrary unit.

Next, we evaluated the effect of AHE and agerarin on IL-4-induced *PER2* oscillation. HaCaT/PER2:luc2P bioluminescence reporter cells were synchronized with 100 nM dexamethasone for 2 h and treated with AHE or agerarin, after which the phase, period, and amplitude parameters of real-time PER2:luc2P bioluminescence were analyzed. We observed that IL-4 shortened the phase (Figure 7A), lengthened the period (Figure 7B), and enhanced the peak amplitude (Figure 7C) of PER2:luc2P bioluminescence compared to the control. Treatment with AHE or agerarin restored the IL-4-induced alteration of PER2:luc2P oscillations to a pattern close to that of the control. These findings suggest that agerarin in AHE could restore the altered rhythmic expression of *PER2* induced by IL-4, possibly through the downregulation of *PER2* expression.

## 3. Discussion

In this study, we demonstrated that IL-4 enhanced *PER2* expression through activation of the JAK/STAT3 signaling pathway and the amplitude of the circadian oscillation of *PER2* promoter activity in HaCaT keratinocytes. Agerarin is an effective natural JAK1/2 inhibitor [28], and we found that it alleviated IL-4-induced *PER2* expression and restored the oscillation of PER2 expression altered by IL-4 stimulation.

Inflammation is a complex biological response of body tissues to harmful stimuli, such as pathogens and chemical irritants, to eliminate the initial cause of tissue damage and repair damaged tissues. However, prolonged inflammation is an important pathogenic feature and is closely associated with various diseases, such as AD and osteoarthritis. Multiple immune cells, including dendritic cells, T lymphocytes, macrophages, and mast cells, are involved in the inflammatory response. Activated CD4^+^ Th cells differentiate into two major subtypes: Th1 cells, which lead to a cell-mediated response, and Th2 cells, which lead to a humoral immune response [34]. Th2 cells produce effector cytokines, such as IL-4, IL-5, IL-6, and IL-13. In particular, IL-4 plays an autoregulatory role in promoting Th2 cytokine production and stimulates B cells to produce immunoglobulin E, which, in turn, stimulates mast cells to release histamine and leukotriene to cause allergic reactions [35]. In addition to its effect on immune cells, IL-4 also acts on keratinocytes to induce thymic stromal lymphopoietin production, which is involved in the maturation of T cell populations and epidermal Langerhans cells [36]. Therefore, IL-4 signaling is considered a potential therapeutic target for AD treatment [37].

Mammalian circadian rhythms are endogenous clocks that help organisms adapt to daily changes in response to environmental stimuli. The SCN functions as a master circadian oscillator that controls several physiological responses, such as the sleep cycle, endocrine system, heart rate, metabolism, and immune function [38,39]. Several studies have shown that circadian clock components are involved in skin biology, including hydration and skin aging. CLOCK regulates the expression of not only AQP3, which regulates hydration by transporting water and glycerol in the skin [39] but also tissue inhibitor of the metalloproteinase 3 gene in HaCaT keratinocytes [40]. Meanwhile, PERIOD is involved in skin aging by mediating MMP-1 gene expression [41].

Persistent loss of sleep disrupts the circadian rhythm. It is well established that constant disruption of the circadian rhythm increases the incidence rate of chronic diseases, such as diabetes, obesity, AD, and cancer [12,42,43,44]. Immune function is also controlled by circadian rhythm, including the number and function of circulating leukocytes and types of cytokine production [45,46]. In the present study, we observed that IL-4 stimulation stimulated the JAK-STAT3 pathway and caused a significant increase in the mRNA expression of *PER2*. In addition, inhibition of JAK kinase activity with the JAK2 inhibitor AG490 or a pan-JAK inhibitor pyridone suppressed IL-4-induced PER2 expression. In addition, *STAT3* knockdown abrogated IL-4-induced PER2 expression. Furthermore, IL-4 altered oscillatory *PER2* promoter activity in HaCaT keratinocytes. These data suggest that the JAK-STAT3 signaling pathway links inflammatory cytokine signaling and the circadian clock system in HaCaT keratinocytes. Thus, targeting the JAK/STAT pathway may be a potential therapeutic approach for the treatment of inflammatory skin disorders associated with disrupted circadian rhythms, such as AD.

Does the enhanced expression of PER2 contribute to IL-4-mediated inflammatory responses? *Per2* mutant mice exhibited a short period and arrhythmic oscillatory rhythms as compared to wild-type mice [47,48], suggesting that PER2 is necessary for circadian clock rhythm resetting [26] and regulation of the rhythmic period length [27]. Depletion of Cry, which forms a complex with Per2, leads to the elevation of proinflammatory cytokines through constitutive activation of the nuclear factor kappa B and protein kinase A signaling pathways [22]. In addition, the absence of *Per2* alleviated inflammatory responses in an AD animal model [49]. Thus, it is possible that enhanced PER2 expression via the IL-4-mediated JAK/STAT3 pathway might result in the upregulation of various proinflammatory cytokines, thereby contributing to the pathophysiology of skin inflammation, such as AD. However, because STAT3 can also regulate the expression of multiple inflammatory genes in keratinocytes, the precise role of PER2 in skin inflammatory responses remains to be determined.

*A. houstonianum* contains various bioactive components, including flavonoids (agehoustin A, agehoustin B, agehoustin C, agehoustin D, eupalestin, agecorynin C, exoticin, purpurascenin), pyrrolizidine alkaloids (heliohoustine, lycopsamine and retrohoustine), benzofuran, chromenes (precocene I and precocene II), diazoprogesterone, hexadecanoic acid, and squalene [50,51,52,53]. Of these, precocene II, known as agerarin (spectra can be seen at https://static-content.springer.com/esm/art%3A10.1038%2Fs41598-017-11642-x/MediaObjects/41598_2017_11642_ MOESM1_ESM.pdf) (accessed on 1 May 2022), is a natural selective inhibitor of JAK1 (IC50, 0.473 μM) with a weak inhibitory activity against JAK2 and 3 (IC50, 4.92 and 3.12 μM, respectively) but not TYK2 [49]. Here, we also observed that the JAK2 inhibitor AG490 and pan-JAK inhibitor pyridone restored PER2 expression enhanced by IL-4. Thus, we speculated that agerarin could prevent IL-4-induced PER2 expression. To test this possibility, we first confirmed the inhibitory effect of agerarin on the IL-4-induced JAK-STAT3 pathway. Previously, we reported that AHE induced AQP3 expression at a concentration of 5–20 µg/mL in a dose-dependent manner [29], while agerarin inhibited TNFα+IFNγ-induced phosphorylation of JAK1, JAK2, and STAT3 at concentrations above 10 µM in a dose-dependent manner in HaCaT keratinocytes [28]. Therefore, in the present study, we confirmed the inhibitory effects of AHE at 10 and 20 µg/mL concentrations and agerarin at 10 and 20 µM concentrations on the JAK-STAT3 pathway. Consistent with a previous study [28], AHE and agerarin dose-dependently inhibited IL-4-induced phosphorylation of JAK1 and JAK2, as well as that of STAT3 at Tyr-705, a downstream target of JAK1/2. Under these experimental conditions, we tested whether agerarin prevented PER2 expression induced by IL-4 and found that it reduced PER2 mRNA and protein levels. Furthermore, agerarin restored the oscillatory rhythmicity of PER2 expression altered by IL-4. These data suggest that the JAK-STAT3 axis but not TYK2 is critical for PER2 overexpression by IL-4 and that inhibition of the JAK-STAT3 pathway may be an effective strategy for the restoration of disturbed circadian clock systems induced by aberrantly expressed PER2. It has also been reported that agerarin downregulated STAT3 expression in melanocytes and is known to inhibit phosphorylation of JAK1/2 and STAT3 by IL-4 plus IL-13 and TNFα plus IFNγ in HaCaT keratinocytes [28,54]. These findings suggest that the natural JAK inhibitor agerarin may help in alleviating the altered circadian rhythm of keratinocytes during skin inflammation. However, we cannot rule out the possibility that agerarin exerts an off-target effect in addition to JAK kinases, leading to the downregulation of PER2 expression independent of the JAK-STAT3 pathway. In this regard, further detailed studies are necessary to determine whether agerarin affects the expression of other circadian clock genes and whether it can restore the disturbed circadian rhythms of keratinocytes and immune cells during inflammation.

In summary, the current study showed that: (i) IL-4 upregulates PER2 expression at the transcriptional level; (ii) the JAK/STAT3 pathway is involved in IL-4-induced PER2 upregulation; and (iii) the natural JAK inhibitor agerarin restores PER2 expression and its oscillatory rhythmicity altered by IL-4. Thus, agerarin targeting the JAK/STAT pathway may be useful as a potential cosmeceutical agent against inflammatory skin diseases associated with disrupted circadian rhythms, such as AD.

## 4. Materials and Methods

### 4.1. Materials

The ethanolic extract of *Ageratum houstonianum* (AHE) was prepared as described previously [29]. Human IL-4 was obtained from Peprotech (Rocky Hill, NJ, USA). Primary antibodies against phospho-JAK1 (Y1034/1035), total JAK1, phospho-JAK2 (Y1007/1008), total JAK2, phospho-STAT3 (Y705), and total STAT3 were purchased from Cell Signaling Technology (Beverly, MA, USA). Primary antibodies specific to glyceraldehyde 3-phosphate dehydrogenase (GAPDH) and PER2 were purchased from Santa Cruz Biotechnology (Santa Cruz, CA, USA). Agerarin (CAS number: 644-06-4) and other chemical reagents were obtained from Sigma-Aldrich (St. Louis, MO, USA).

### 4.2. Cells and Cell Culture

Human keratinocyte HaCaT cells were obtained from the Cell Line Service (Eppelheim, Germany). The cells were cultured in Dulbecco’s modified Eagle’s medium supplemented with 10% fetal bovine serum (HyClone, Logan, UT, USA) and penicillin-streptomycin (Sigma-Aldrich).

### 4.3. RT-PCR

Total RNA was isolated from HaCaT cells using a TRIzol RNA extraction kit (Invitrogen, Carlsbad, CA, USA) and was reverse transcribed to cDNA using an iScript cDNA synthesis kit (Bio-Rad, Hercules, CA, USA). RT-PCR was performed using reverse transcriptase (Promega, Madison, WI, USA) and gene-specific PCR primers as follows:

- PER2 forward, 5′-CCA CGA GAA TGA AAT CCG CT-3′;
- PER2 reverse, 5′-CCT CCC AAT GAT GAA GGA GA-3;
- GAPDH forward, 5′-ACC CAC TCC TCC ACC TTT G-3′;
- GAPDH reverse, 5′-CTC TTG TGC TCT TGC TGG G-3′.

The thermal cycling conditions were as follows: denaturation at 94 °C for 5 min, followed by 30 cycles of denaturation at 94 °C for 30 s, annealing at 54.5 °C for 30 s, and elongation at 72 °C for 1 min. The amplified PCR products were separated by electrophoresis on a 1% agarose gel containing ethidium bromide and visualized under UV transillumination.

### 4.4. Quantitative Real-Time PCR (qPCR)

Total RNA was isolated as described previously. qPCR was performed on an iCycler iQ system with an iQ SYBR Green Supermix kit (Bio-Rad). Validated commercial qPCR primers and SYBR Green-based fluorescent probes specific for *PER2* (id: qHsaCEP0051693) and *GAPDH* mRNA (id: qHsaCEP0041396) were obtained from Bio-Rad. The thermal cycling conditions for PCR were as follows: denaturation at 95 °C for 2 min, followed by 40 cycles of denaturation at 95 °C for 10 s and annealing at 60 °C for 45 s. The relative expression of mRNA was normalized to that of GAPDH using the software provided by the manufacturer.

### 4.5. Immunoblot Analysis

HaCaT cells were lysed in ice-cold cell lysis buffer supplemented with 20 mM hydroxyethyl piperazineethanesulfonic acid (pH 7.2), 1% Triton X-100, 150 mM NaCl, 10% glycerol, 1 mM EDTA, 1 mM Na_3_VO_4_, 1 mM NaF, 10 μg/mL leupeptin, and 1 mM phenylmethylsulfonyl fluoride. The proteins were separated by electrophoresis on an 8% sodium dodecyl sulfate-polyacrylamide gel and transferred to nitrocellulose membranes. The membranes were blocked with 5% skimmed milk for 30 min and then incubated with the appropriate primary antibodies for 4 h at 25 °C and secondary antibodies overnight at 4 °C. The immunoreactive protein bands were visualized on a CP-BU X-ray film (Agfa, European communities) using an enhanced chemiluminescence detection system (GE Healthcare, Piscataway, NJ, USA). In some experiments, the relative intensities of the immunoreactive bands were measured using ImageJ version 1.52a (National Institutes of Health, Bethesda, MD, USA).

### 4.6. Immunofluorescence Microscopic Analysis

Immunofluorescent staining of HaCaT cells was performed as previously described [28]. Briefly, HaCaT cells seeded on coverslips were treated with vehicle (phosphate-buffered saline; PBS), 20 ng/mL IL-4, or 20 ng/mL IL-4 in the presence of 40 μM agerarin. After 24 h, the cells were fixed with 3.7% paraformaldehyde for 10 min and permeabilized with 0.3% Triton X-100 for 15 min. After blocking with 5% bovine serum albumin for 1 h, the cells were incubated with primary antibodies specific to αβ-tubulin (1:100) and PER2 (1:100) for 2 h, followed by the addition of Alexa Fluor 488-conjugated (1:300; green fluorescence for αβ-tubulin) or Alexa Fluor 555-conjugated (1:300; red fluorescence for PER2) secondary antibodies (Invitrogen) for 40 min at 25 °C. Nuclear DNA was stained with 1 μg/mL Hoechst 33258 (blue fluorescence) for 10 min. Fluorescent cells were captured using an EVOS FL fluorescence microscope (Advanced Microscopy Group, Bothell, WA, USA).

### 4.7. Construction of Human PER2 Promoter-Reporter and Generation of Real-Time Bioluminescence Reporter Cells

A *PER2* promoter fragment spanning nucleotides −500 to +50 upstream of the transcription start site was synthesized from human genomic DNA (Promega) via PCR with the primers 5′-ggG cgt agt gaa tgg aag gcg-3′ and 5′-cag cag ccc aag gaa ctt-3′. The PCR products were ligated into a T&A vector (RBC Bioscience, Taipei County, Taiwan), followed by enzyme digestion with *Kpn*I and *Bgl*II and ligation into the corresponding sites of the pGL4.2(luc2P/minP/Hygro) vector (Promega), yielding the pPER2:luc2P bioluminescence reporter. HaCaT cells were transfected with pPER2:luc2P using the Nucleofector electroporation kit (Amaxa, Inc., Köln, Germany), followed by selection with 10 μg/mL hygromycin (Sigma-Aldrich). After four weeks, a stable bioluminescence reporter cell line (HaCaT/PER2:luc2P) was obtained.

### 4.8. Real-Time Bioluminescence Analysis

For real-time bioluminescence monitoring, HaCaT/PER2:luc2P reporter cells were seeded to white 96-well plates (Thermo Fisher Life Sciences, Middlesex, MA, USA) at a density of 2.0 × 10^4^ cells/well. After 2 d, the cells were synchronized with 100 µM dexamethasone for 2 h, after which the medium was replaced with a phenol red-free recording medium containing 1% fetal bovine serum, 1% penicillin/streptomycin, 0.2 mg/mL hygromycin, and 1 mM D-luciferin (Promega). The plates were sealed, and bioluminescence was continuously recorded at intervals of 20 min for 5 d using Spark10M (Tecan, Männedorf, Switzerland). Circadian parameters (period, phases, and amplitude) were analyzed using Biodare2 (www.biodare2.ed.ac.uk) (accessed on 3 June 2021).

### 4.9. Statistical Analysis

Data are expressed as the mean ± standard deviation. Statistical analysis was performed using a one-way analysis of variance, followed by Dunnett’s or Sidak’s multiple comparisons test using GraphPad Prism (version 9.2.0; GraphPad Software, Inc., La Jolla, CA, USA). Statistical significance was set at *p* < 0.05.

## 5. Conclusions

This study demonstrates that the Th2 cytokine IL-4 upregulates PER2 expression by activating the JAK/STAT3 signaling pathway, and a natural JAK inhibitor, agerarin, reduces PER2 expression induced by IL-4. Furthermore, agerarin restores the circadian rhythmicity of PER2 expression altered by IL-4 in HaCaT keratinocytes. These findings suggest that agerarin can be used as a beneficial compound for cosmeceutical applications against inflammatory skin conditions associated with disrupted circadian rhythms, such as AD.

## Data Availability

Not applicable.

## Supplementary Materials

The following supporting information can be downloaded at: https://www.mdpi.com/article/10.3390/molecules27134205/s1. Supporting Information S1–S8, Uncropped gels and blots; Supplementary Figure S1, Generation of live-cell bioluminescence reporter for monitoring of *PER2* expression in live cells.
