# Supplementary material for: The Natural Janus Kinase Inhibitor Agerarin Downregulates Interleukin-4-Induced PER2 Expression in HaCaT Keratinocytes
Source: Molecules. 2022 Jun 30;27(13):4205. doi: 10.3390/molecules27134205 (PMC9268509; doi:10.3390/molecules27134205)
Supplement: Supplementary file 1 [file molecules-27-04205-s001.zip › molecules-1697424-supplementary.pdf]

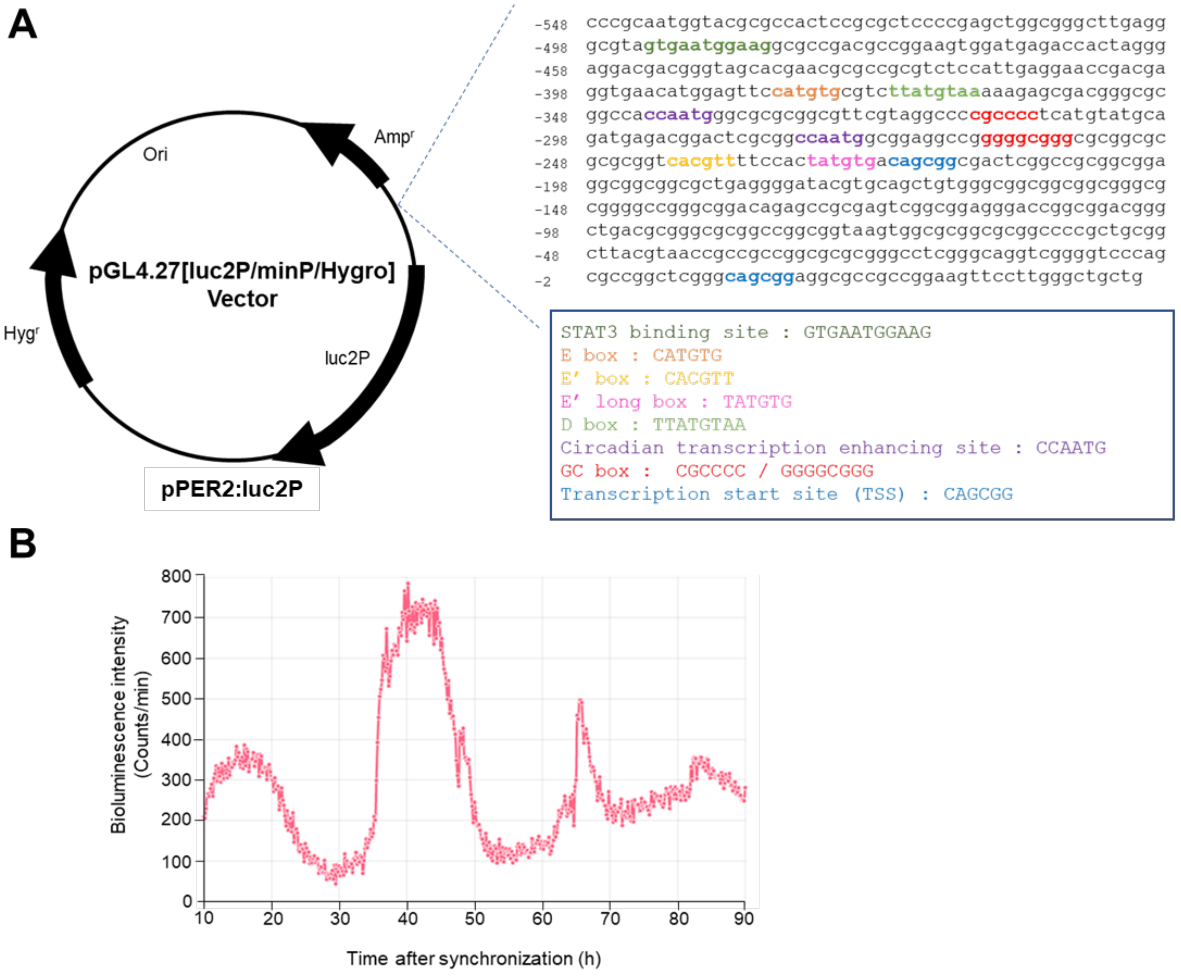

Supplementary Figure S1. Generation of live-cell bioluminescence reporter for monitoring of *PER2* expression in live cells. (A) Construction of pPER2:luc2P reporter contained the 5'-regulatory region of the *PER2* gene (-500/+50). STAT3 binding site (SBS), E-box, E'-box, D-Constructionbox, circadian transcription enhancing site (CTIES), GC-box, and transcription initiation site (TIS) are underlined. (B) Representative trace of bioluminescence of PER2:luc2P reporter. HaCaT/PER2:luc2P cells seeded to 96-well plates were synchronized with 100 µM dexamethasone for 2 h, then changed to the recording medium containing 1% fetal bovine serum and 1 mM luciferin in a phenol-free medium. Real-time bioluminescence of PER2:luc2P reporter was measured and recorded every 20 min for up to 90 h using Spark10M.
