# Supplementary material for: The Natural Janus Kinase Inhibitor Agerarin Downregulates Interleukin-4-Induced PER2 Expression in HaCaT Keratinocytes
Source: Molecules. 2022 Jun 30;27(13):4205. doi: 10.3390/molecules27134205 (PMC9268509; doi:10.3390/molecules27134205)
Supplement: Supplementary file 1 [file molecules-27-04205-s001.zip › molecules-1697424-Supporting Information_Uncropped blots.pdf]

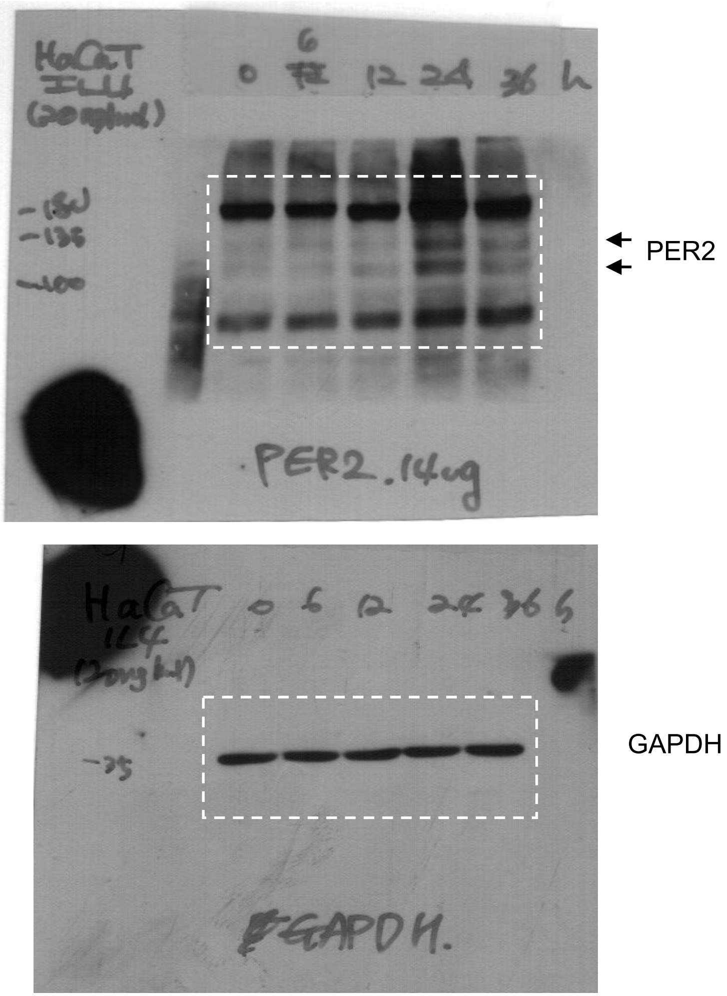

Supporting Information S1. Uncropped blots in Figure 1C.

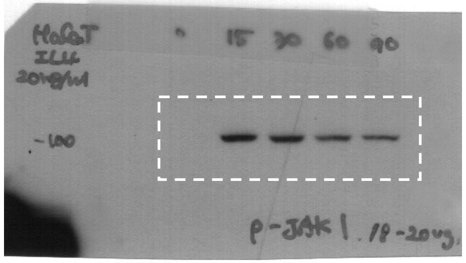

p-JAK1
(Y1034/1035)

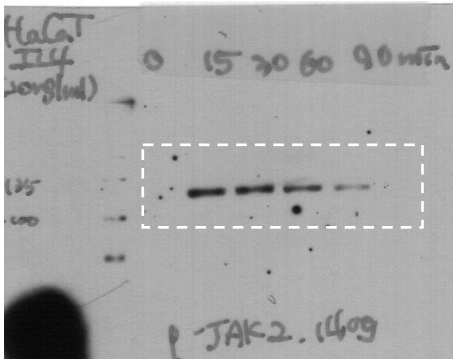

p-JAK2
(Y1007/1008)

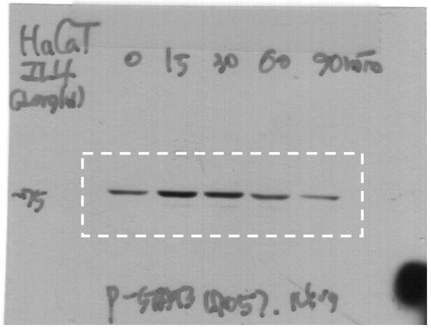

p-STAT3
(Y705)

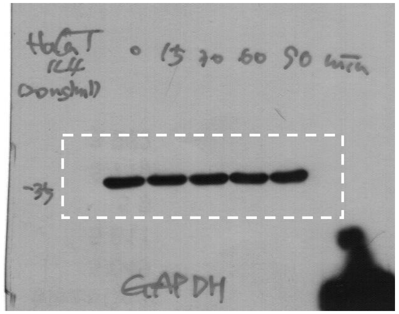

GAPDH

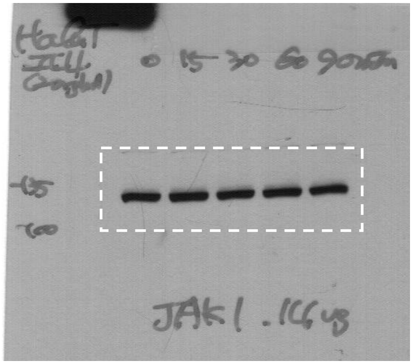

JAK1

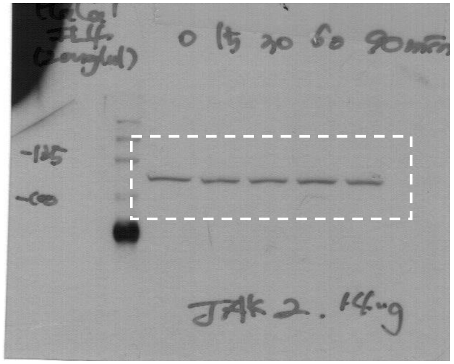

JAK2

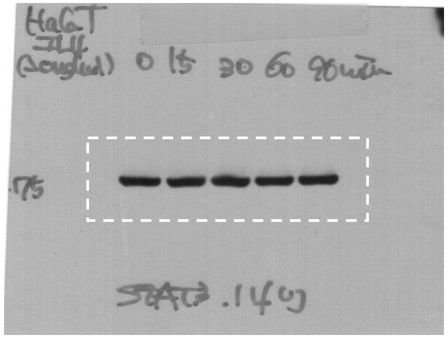

STAT3

Supporting Information S2. Uncropped blots in Figure 2A.

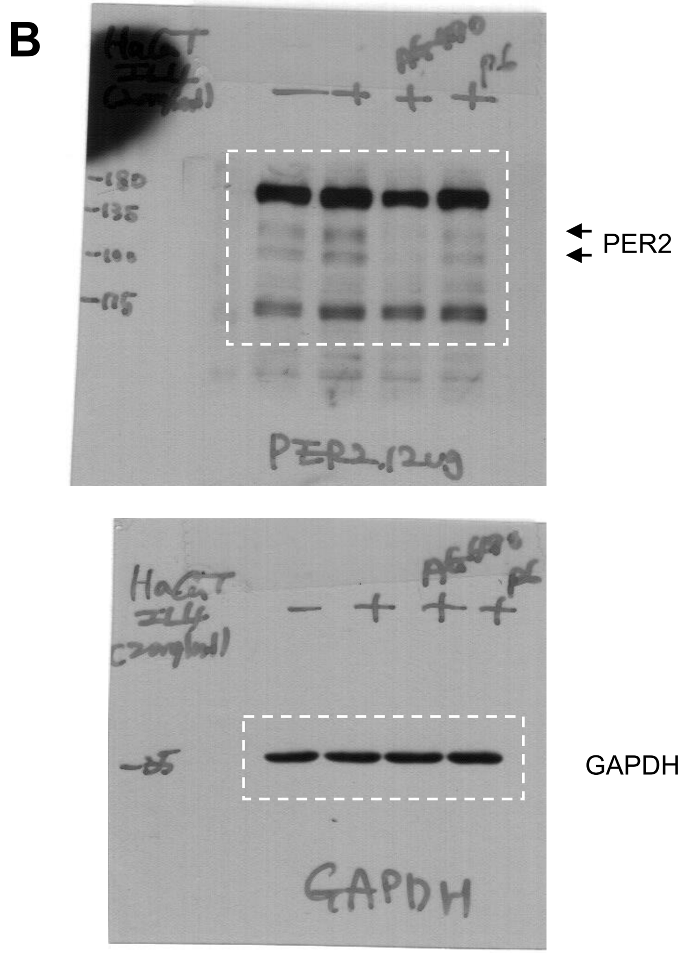

Supporting Information S3. Uncropped blots in Figure 2B.

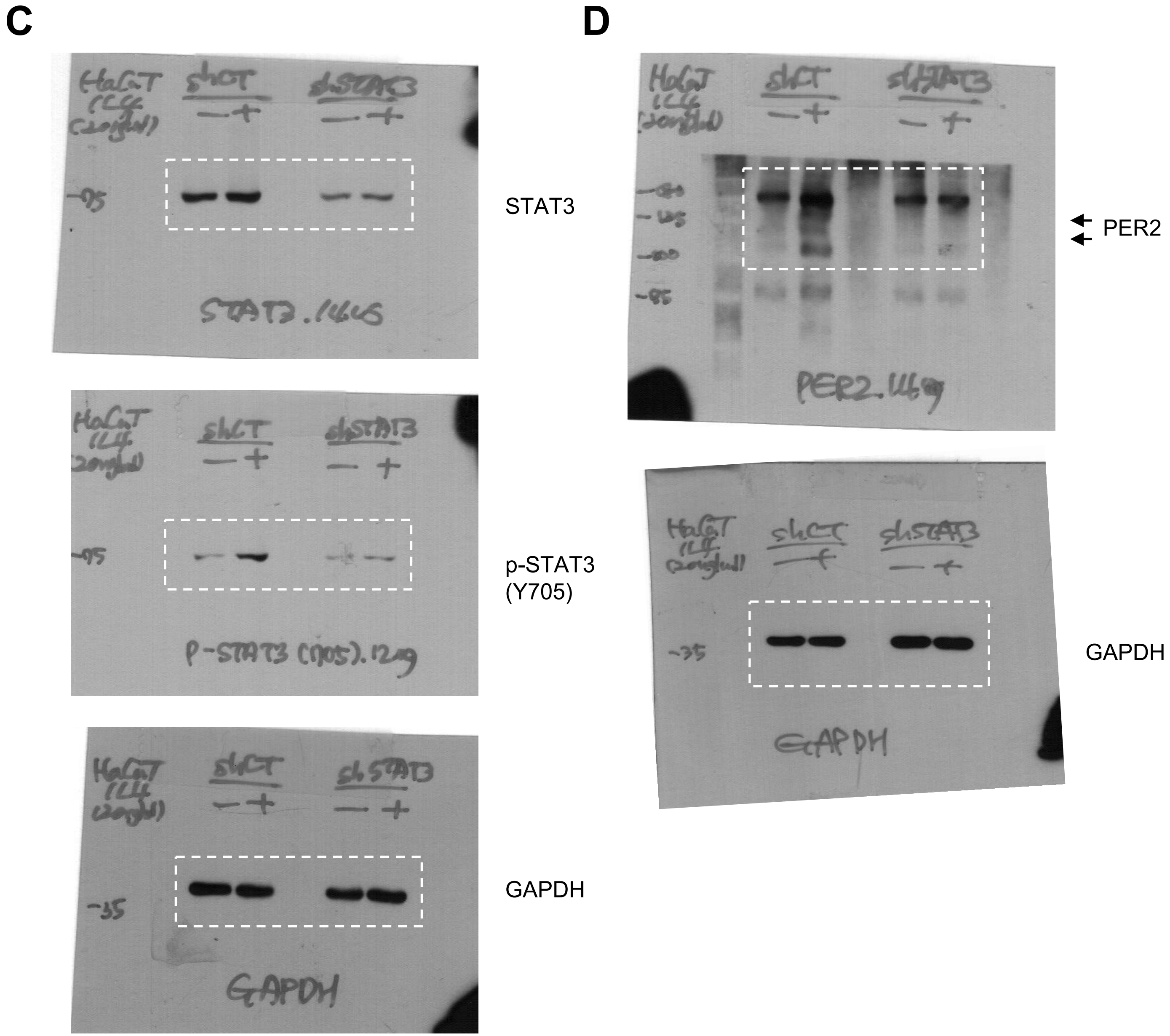

Supporting Information S4. Uncropped blots in Figure 2C and D.

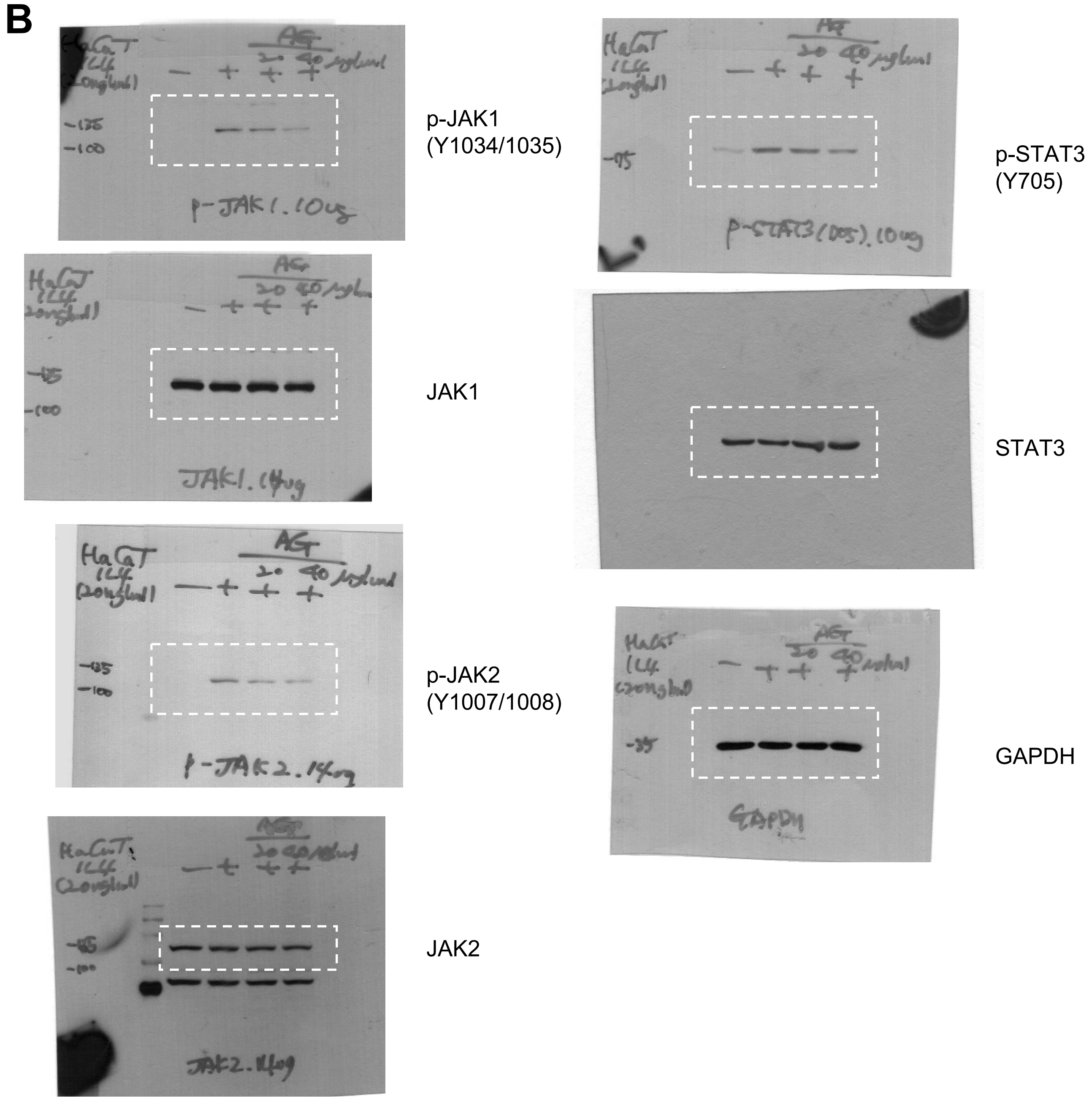

Supporting Information S5. Uncropped blots in Figure 3B.

C

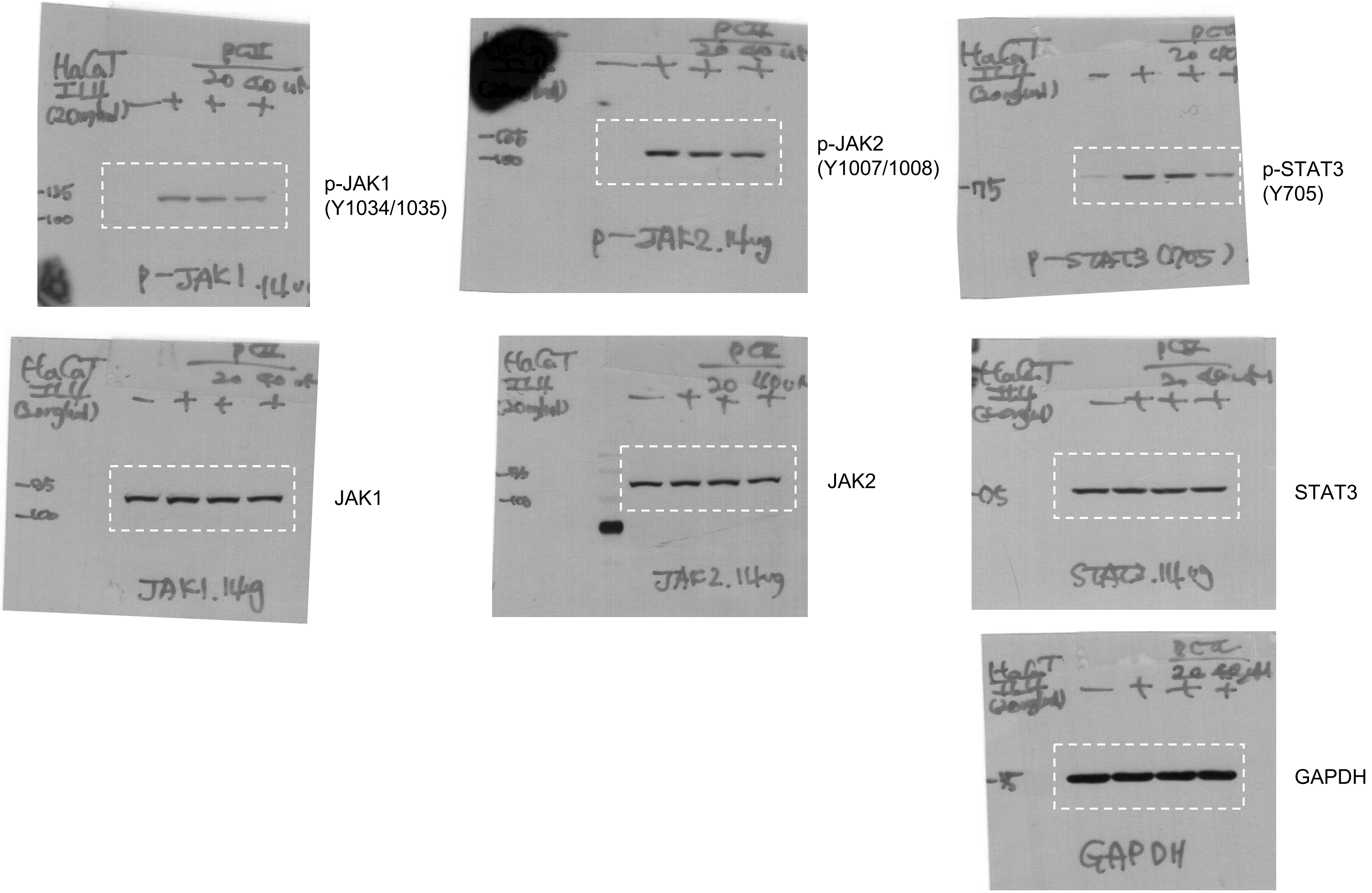

Supporting Information S6. Uncropped blots in Figure 3C.

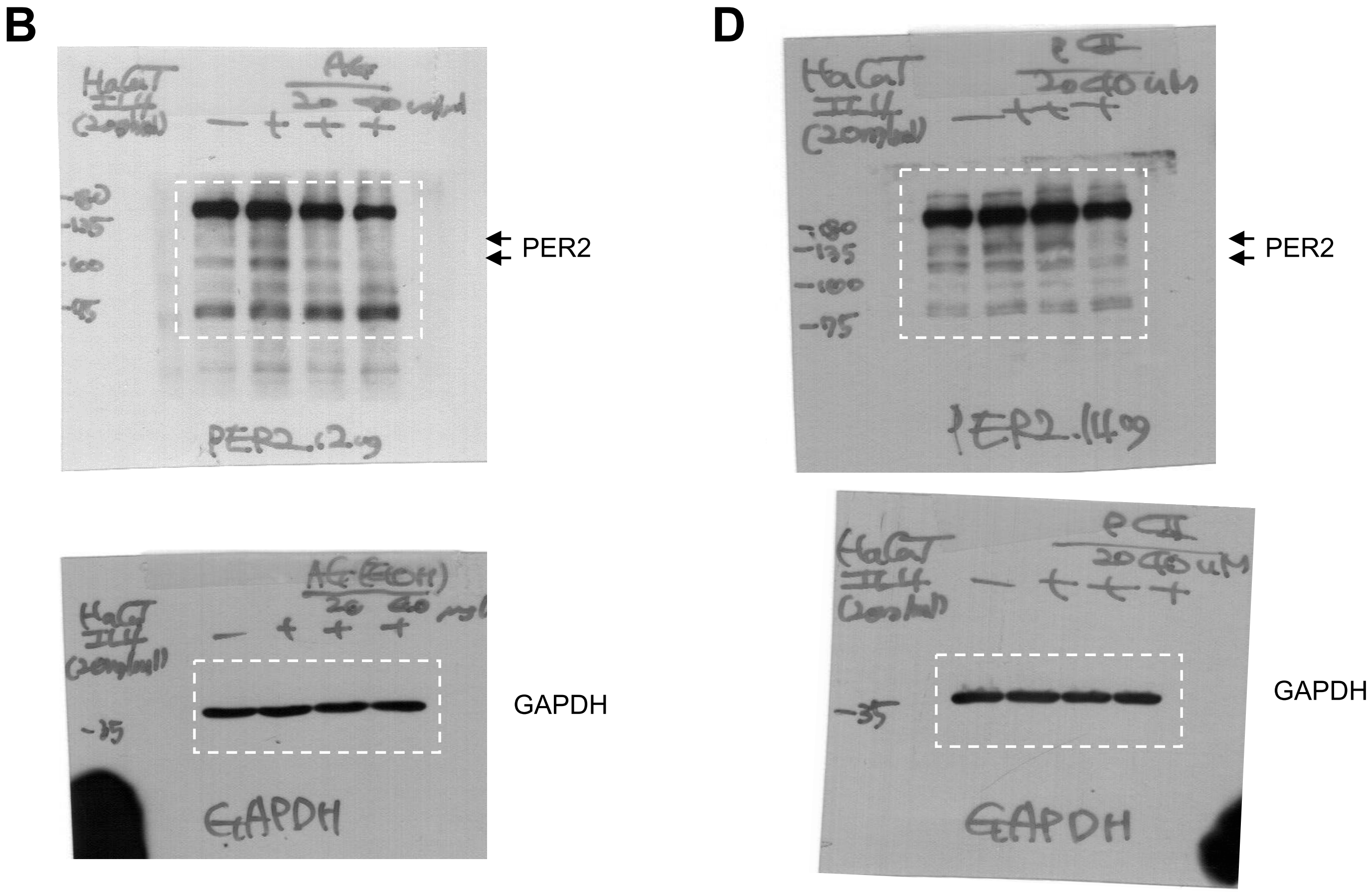

Supporting Information S7. Uncropped blots in Figure 4B and D.

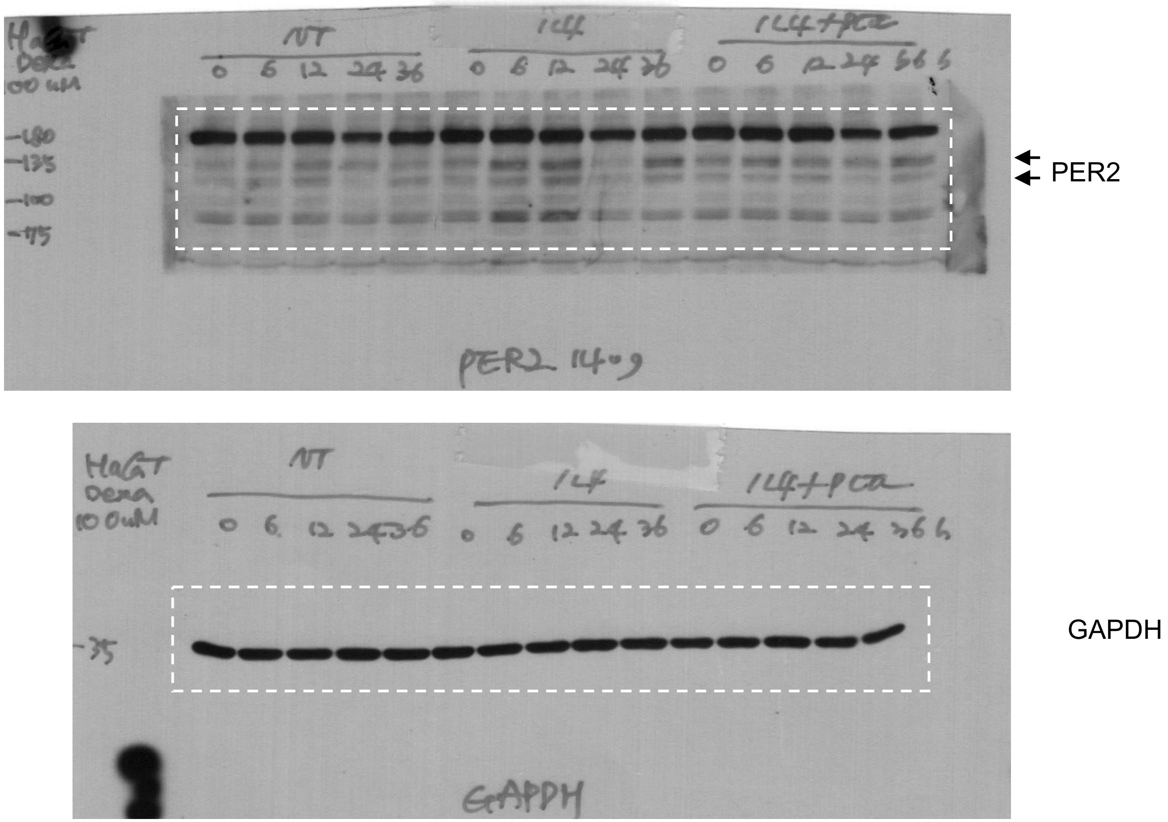

Supporting Information S8. Uncropped blots in Figure 6.
